# Seroprevalence of Influenza A(H1N1)pdm09 Virus Antibody, England, 2010 and 2011

**DOI:** 10.3201/eid1811.120720

**Published:** 2012-11

**Authors:** Katja Hoschler, Catherine Thompson, Nick Andrews, Monica Galiano, Richard Pebody, Joanna Ellis, Elaine Stanford, Marc Baguelin, Elizabeth Miller, Maria Zambon

**Affiliations:** Health Protection Agency, London, UK (K. Hoschler, C. Thompson, N. Andrews, M. Galiano, R. Pebody; J. Ellis, M. Baguelin, E Miller, M. Zambon);; and Health Protection Agency, Manchester, UK (E. Stanford)

**Keywords:** influenza, influenza virus, influenza A(H1N1)pdm09 virus, serology, seroprevalence, antibody, pandemic, incidence, transmission, antigenic drift, vaccination, severity, surveillance, viruses, England

## Abstract

The intense influenza activity in England during the 2010–11 winter resulted from a combination of factors. Population-based seroepidemiology confirms that the third wave of influenza A(H1N1)pdm09 virus circulation was associated with a shift in age groups affected, with the highest rate of infection in young adults.

Seroepidemiologic data collected in England during the first 2 influenza pandemic waves suggested that another wave of infection with influenza A(H1N1)pdm09 virus was unlikely during 2010–11 (*1*). However, a substantial third wave occurred that affected persons in older age groups (*2*). Severity indicators suggested a higher level of illness and death, with increased cases in critical care and deaths. We conducted further seroepidemiologic study in England during 2010–11 to identify possible reasons for these observations.

## The Study

This observational study used anonymized, residual serum samples from routine microbiological testing. Patient age and sex, date of sample collection, and source laboratory information were available (*3*).

**Figure 1:**
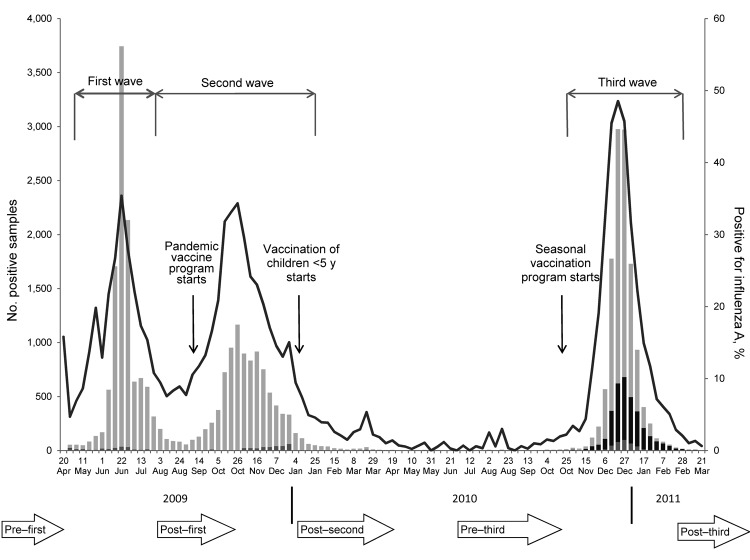
Number of influenza A(H1N1)pdm09 virus detections (and percentage positive) detected through a network of Health Protection Agency laboratories (the Respiratory DataMart system) from the start of the pandemic in week 17 (week of April 27) 2009 until the end of the 2010–11 winter season. It demonstrates the 3 waves of pandemic influenza activity in summer 2009, autumn 2009, and winter 2010–11 and the key events in relation to the timing of the national influenza vaccination program. The timing of the serum collections are illustrated at the bottom of the figure. Samples were grouped into panels according to their collection. Light gray, A(H1N1)pdm09 virus; medium gray, untyped influenza A virus; black, influenza B virus; line, overall percentage positive. Study periods were defined as follows: pre–first wave, before April 2009; post–first wave, August–October 2009; post–second wave, January–April 2010; pre–third wave, June–October 2010; and post–third wave, February–April 2011.

Samples were from patients 0–99 years of age, of whom 53% were female. Samples were grouped according to collection date: pre–first wave (before April 2009 [1,403 samples]) and post–first wave (August–October 2009 [3,091 samples]); post–second wave (January–April 2010 [2,225 samples]); and pre–third wave (June–October 2010 [1,782 samples]) and post–third wave (February–April 2011 [1,257 samples]) (Figure 1). Availability of samples by region and patient age was not consistent. With the objective of measuring age-dependent incidence, we prioritized serum samples by patient age. Samples were spread across 7 age groups (<5, 5–14, 15–24, 25–44, 45–64, 65–74, and >75 years) and came from the 9 regions of England (East, East Midlands, London, North East, North West, South East, South West, West Midlands, and Yorkshire and Humber).

Viruses were characterized and sequenced as described (*4*). All samples were tested by hemagglutination-inhibition (HI) assay; samples with sufficient material also were tested by microneutralization assay according to standard methods (*1*). Samples with titers >32 or >40 by HI or microneutralization assay, respectively, were considered seropositive.

We determined antibody persistence by comparing antibody levels in the post–second wave panel with those of the pre–third wave panel on a subset of samples from 3 regions (North East, North West, and South West) where samples were available for both time points. Results were assessed with 95% confidence intervals. The full analysis of the seroprevalence preceding the 2010–11 season is detailed elsewhere (*1*).

**Figure 2:**
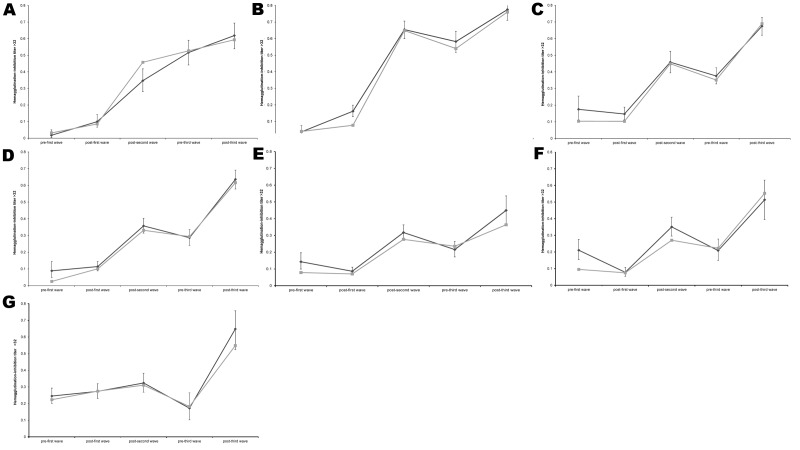
Percentage of samples with hemagglutination-inhibition titer >32 during consecutive waves of influenza activity, England, summer 2009 and 2009-10 and 2010-11 influenza seasons. Data were plotted from all available results determined by hemagglutination-inhibition assay on samples from all regions. A) Children <5 years old. B) Children 5–14 years old. C) Persons 15–24 years old. D) Persons 25–44 years old. E) Persons 45–64 years old. F) Persons 65–74 years old. G) Persons >75 years old. Black line, results from all regions; gray line, results from the North West and South West regions, which provided samples throughout the entire period. Error bars indicate 95% confidence intervals.

In samples from all persons except those in the youngest age group (<5 years), antibody declined from the end of the 2009–10 winter season (post–second wave) to before the onset of the 2010–11 season (pre–third wave). This decline was limited (<10% reduction by HI and microneutralization assays in persons 5–74 years of age), with the largest reduction in the >75-year group (−15% and −20% by HI and microneutralization assays, respectively). In children <5 years, antibody levels increased (15% and 10% by HI and nicroneutralization assays, respectively) during the same time period (Table 1, Table 2; Figure 2).

**Table 1:** Seroprevalence of influenza A(H1N1)pdm09 virus antibody, England, 2010 and 2011*

Characteristic	No. seropositive samples/no. total samples† (% seropositive samples, 95% CI)							
	Post–second wave‡			Pre–third			Post–third wave	
	HI	MN		HI	MN		HI	MN
Age group, y								
<5	36/98 (0.37, 0.27–0.47)	31/77 (0.4, 0.29–0.52)		94/182 (0.52, 0.44–0.59)	88/174 (0.51, 0.43–0.58)		99/160 (0.62, 0.54–0.69)	93/150 (0.62, 0.54–0.7)
5–14	132/213 (0.62, 0.55–0.69)	69/107 (0.64, 0.55–0.73)		142/244 (0.58, 0.52–0.64)	152/237 (0.64, 0.58–0.7)		155/200 (0.78, 0.71–0.83)	146/199 (0.73, 0.67–0.79)
15–24	68/154 (0.44, 0.36–0.52)	44/101 (0.44, 0.34–0.54)		152/405 (0.38, 0.33–0.42)	156/400 (0.39, 0.34–0.44)		216/320 (0.68, 0.62–0.73)	188/311 (0.6, 0.55–0.66)
25–44	66/200 (0.33, 0.27–0.4)	31/83 (0.37, 0.27–0.49)		106/370 (0.29, 0.24–0.34)	114/370 (0.31, 0.26–0.36)		187/294 (0.64, 0.58–0.69)	155/283 (0.55, 0.49–0.61)
45–64	59/220 0.27, 0.21–0.33)	42/110 (0.38, 0.29–0.48)		69/320 (0.22, 0.17–0.26)	93/318 (0.29, 0.24–0.35)		62/138 (0.45, 0.36–0.54)	52/135 (0.39, 0.3–0.47)
65–74	36/145 (0.25, 0.18–0.33)	27/87 (0.31, 0.22–0.42)		35/168 (0.21, 0.15–0.28)	42/167 (0.25, 0.19–0.32)		38/74 (0.51, 0.39–0.63)	38/74 (0.51, 0.39–0.63)
>75	55/172 (0.32, 0.25–0.4)	77/163 (0.47, 0.39–0.55)		16/93 (0.17, 0.1–0.26)	25/92 (0.27, 0.18–0.37)		46/71 (0.65, 0.53–0.76)	39/71 (0.55, 0.43–0.67)
Region§								
Total	1,202			1,782			1,257	
North West	561			624			337	
South West	404			232			265	
North East	237			526			179	
East	0			292			122	
Yorkshire and Humber	0			108			354	

*HI, hemagglutination inhibition assay; MN, microneutralization assay. †Samples with titers >32 by HI or >40 by MN, ‡For data consistency, samples from only North West, South West, and North East regions were included in this analysis because these regions also were among the 5 regions in pre– and post–third wave samples. §Numbers by region describe total available number of samples (all analyzed at least by HI assay).

**Table 2:** Seroincidence estimates of influenza A(H1N1)pdm09 virus antibody, England, 2010 and 2011

Age group, y	Change in antibody level, % (95% CI)				
	Post–second wave to pre–third wave*			Pre–third wave to post–third wave†	
	Hemagglutination inhibition	Microneutralization		Hemagglutination inhibition	Microneutralization
<5	15 (3–27)	10 (–3 to 24)		10 (0–21)	11 (1–22)
5–14	–4 (–13 to 5)	0 (–11 to 11)		19 (11–28)	9 (1–18)
15–24	–7 (–16 to 3)	–5 (–15 to 6)		30 (23–37)	21 (14–29)
25–44	–4 (–12 to 4)	–7 (–18 to 5)		35 (28–42)	24 (16–31)
45–64	–5 (–13 to 2)	–9 (–19 to 1)		23 (15–33)	9 (0–19)
65–74	–4 (–13 to 5)	–6 (–18 to 6)		31 (18–43)	26 (13–39)
>75	–15 (–25 to –4)	–20 (–32 to –8)		48 (34–61)	28 (13–42)

*Antibody levels at the end of the 2009–10 winter season compared with those before the 2010–11 season. †Antibody levels before and after the 2010–11 season.

We assessed changes in antibody levels during the 2010–11 season using data from all 5 available regions (East, North East, North West, South West, and Yorkshire and Humber) (Table 1, Table 2; Technical Appendix Table). For all age groups, HI and microneutralization assays demonstrated similar trends, although the increase by microneutralization assay in elderly persons was lower than by HI assay (48% vs. 28% increase). We found no evidence for association of titer with sex or region.

Children in the 2 youngest groups (<14 years) had the highest titers overall and highest percentage of seropositive samples (Table 1, Table 2; Figure 2; Technical Appendix Table). The highest increases in seroprevalence during the third wave were observed in the oldest age group (>75 years, from 17% to 65% seropositive by HI assay), followed by young adults (15–44 years, from 33% to 66% seropositive by HI assay) (Technical Appendix Figure).

## Conclusions

Clinical surveillance data obtained during the course of acute illness (*2*) and seroepidemiology through population sampling are consistent and together point toward a shift in the age range for infection with A(H1N1)pdm09 in the first season after the 2009 pandemic. This finding is similar to those in earlier pandemics (*5*) and other countries (*6*). Historical data, including from 1918, suggest that the initial impact in children is followed by a dramatic shift in age distribution of infected persons, with the probability of infection in adults exceeding those of children until the age distribution returns to the normal seasonal pattern (*5*,*7*). This adaptation process may take 3–10 years (*7*).

The rates of decline in antibody to A(H1N1)pdm09 from the 2009–10 to the 2010–11 winters are similar to historic data (*8*) and A(H1N1)pdm09 vaccine trials (*9*,*10*). The implications of such reduction are uncertain. The seroprevalence data suggested susceptibility in young adults pre–third wave, but not in children who were targeted by an extended vaccination program in the United Kingdom from January 2010. Up to 30% of children <5 years were vaccinated (*11*).

During the 2010–11 season, antibody was acquired primarily by young and old adults. The largest increase in antibody levels after the 2010–11 winter occurred in persons >75 years of age. Clinical surveillance data suggests that elderly persons (>65 years of age) were relatively spared from infection with A(H1N1)pdm09 virus (*12*). We propose that the increase resulted primarily from seasonal influenza vaccination in 2010–11 with vaccine uptake of 72.8% (*13*). In young adults (15–44 years), we believe that acquisition of antibody occurred as susceptible persons became infected during the winter. Children were relatively spared from infection with A(H1N1)pdm09 during winter 2010–11; their high rate of infection in the 2 previous pandemic waves, together with vaccination, left a limited number of susceptible persons (Figure 2).

Our study design—a retrospective, periodic, cross-sectional collection—has certain limitations. We analyzed similar but not identical groups and persons at different time points. For each sample, only limited information was available. Without information about vaccination status or influenza exposure history during the season, our interpretation of antibody levels and their changes has to be taken with caution. However, in this descriptive analysis we also used supportive evidence from UK influenza surveillance programs and take into account the date of vaccination timing and uptake, which strengthens our interpretation of the serologic data.

The collections for each sample set were distributed over time periods of up to 21 weeks, during which antibody levels would have changed, depending on the combined effects of seroconversion, antibody waning and availability of vaccination. A novel likelihood-based approach, described previously has therefore been developed to overcome some of the limitations of the conventional statistical method (*1*).

We found no evidence of substantial antigenic drift in circulating viruses that could affect seroepidemiology results (Technical Appendix Table). We conclude that the intense A(H1N1)pdm09 virus activity in the England during the 2010–11 winter must have resulted from a combination of factors.

The change in age distribution of infection is likely to have caused increased severity, resulting from a larger number of patients with underlying concurrent conditions (*12*) or from age-dependent changes in pathology. Defining antibody correlates of protection becomes more complex with rising patient age as other immune mechanisms increasingly contribute to protection, e.g., CD4+ T cells, as demonstrated in human challenge experiments (*14*). Moreover, a murine model identified the role of age in susceptibility to pathogenesis and transmission of influenza virus infection (*15*). These observations might help to provide some mechanistic insights for the shift in age distribution of infection and severity in the season after the 2009 pandemic. Genetic drift in circulating virus over time affecting human airway adaptation and varying climatic conditions during different pandemic waves also should be investigated.

Technical Appendix Antibody titers with 2 antigenically divergent influenza A(H1N1)pdm09 viruses and reverse cumulative distribution curves for hemagglutination Inhibition titers post–second wave to pre–third wave, England, 2010 and 2011.
