# Supplementary material for: Seroprevalence of Influenza A(H1N1)pdm09 Virus Antibody, England, 2010 and 2011
Source: Emerg Infect Dis. 2012 Nov;18(11):1894–7. doi: 10.3201/eid1811.120720 (PMC3559155; doi:10.3201/eid1811.120720)
Supplement: Technical Appendix — Antibody titers with 2 antigenically divergent influenza A(H1N1)pdm09 viruses and reverse cumulative distribution curves for hemagglutination Inhibition titers post–second wave to pre–third wave, England, 2010 and 2011. [file 12-0720-Techapp-s1.pdf]

Article DOI: http://dx.doi.org/10.3201/eid1811.120720

Seroprevalence of Influenza A(H1N1)pdm09 Virus Antibody, England, 2010 and 2011

Technical Appendix

Technical Appendix Table. Antibody titers with 2 antigenically divergent influenza A(H1N1)pdm09 viruses, England, 2010 and 2011*

Age group,	A/England/195/09†		A/England/226/10			
	Geometric mean titer (95% CI)		Geometric mean titer (95% CI)		No. with titers >32/no. total samples (p value, 95% CI)	
y	Pre–third wave	Post–third wave	Pre–third wave	Post–third wave	Pre–third wave	Post–third wave
<5	32.6 (24.1–44.1)	52.9 (38.2–73.3)	37 (26.8–51)	60.4 (42.2–86.5)	96/178 (0.54, 0.46–0.61)	91/145 (0.63, 0.54–0.71)
5–14	30.2 (24.3–37.6)	79.6 (62.5–101.5)	37.3 (29.4–47.3)	90.3 (69–118.3)	146/240 (0.61, 0.54–0.67)	141/185 (0.76, 0.69–0.82)
15–24	15.6 (13.2–18.3)	55.2 (45.1–67.7)	15.2 (12.8–17.9)	48.6 (39.3–60.1)	149/389 (0.38, 0.33–0.43)	193/287 (0.67, 0.61–0.73)
25–44	10.1 (8.8–11.6)	41.1 (33.8–50)	10.2 (8.8–11.8)	43.8 (35.9–53.3)	99/357 (0.28, 0.23–0.33)	190/279 (0.68, 0.62–0.74)
45–64	8.8 (7.6–10.2)	22.3 (16.5–30.1)	8.8 (7.5–10.2)	20.8 (15.1–28.6)	70/308 (0.23, 0.18–0.28)	55/127 (0.43, 0.35–0.52)
65–74	8.9 (7.4–10.7)	26 (18–37.7)	7.8 (6.5–9.2)	32.4 (21.9–47.9)	31/159 (0.19, 0.14–0.27)	38/63 (0.6, 0.47–0.72)
≥75	7.9 (6.1–10.2)	47.3 (32.4–69.1)	7.9 (6.1–10.2)	46.6 (30.9–70.2)	15/90 (0.17, 0.1–0.26)	39/59 (0.66, 0.53–0.78)

*From the Pre– and post–third wave panels, samples from 2 regions (North East and North West) were chosen for this experiment; results of their analysis by hemagglutination-inhibition assay were combined for this analysis.
†In experiments with the A/England/195/09 strain, the reverse genetics derivative, NIBRG122 was used.

Technical Appendix Figure (following pages). Reverse cumulative distribution curves for hemagglutination-inhibition titers post–second wave to pre–third wave in a study of influenza A(H1N1)pdm09 virus antibody, England, 2010 and 2011. A) Children <5 years old. B) Children 5–14 years old. C) Persons 15–24 years old. D) Persons 25–44 years old. E) Persons 45–64 years old. F) Persons 65–74 years old. G) Persons >75 years old. Straight line, post–second wave; dashed line, pre–third wave; dotted line, hemagglutination-inhibition titer = 32.

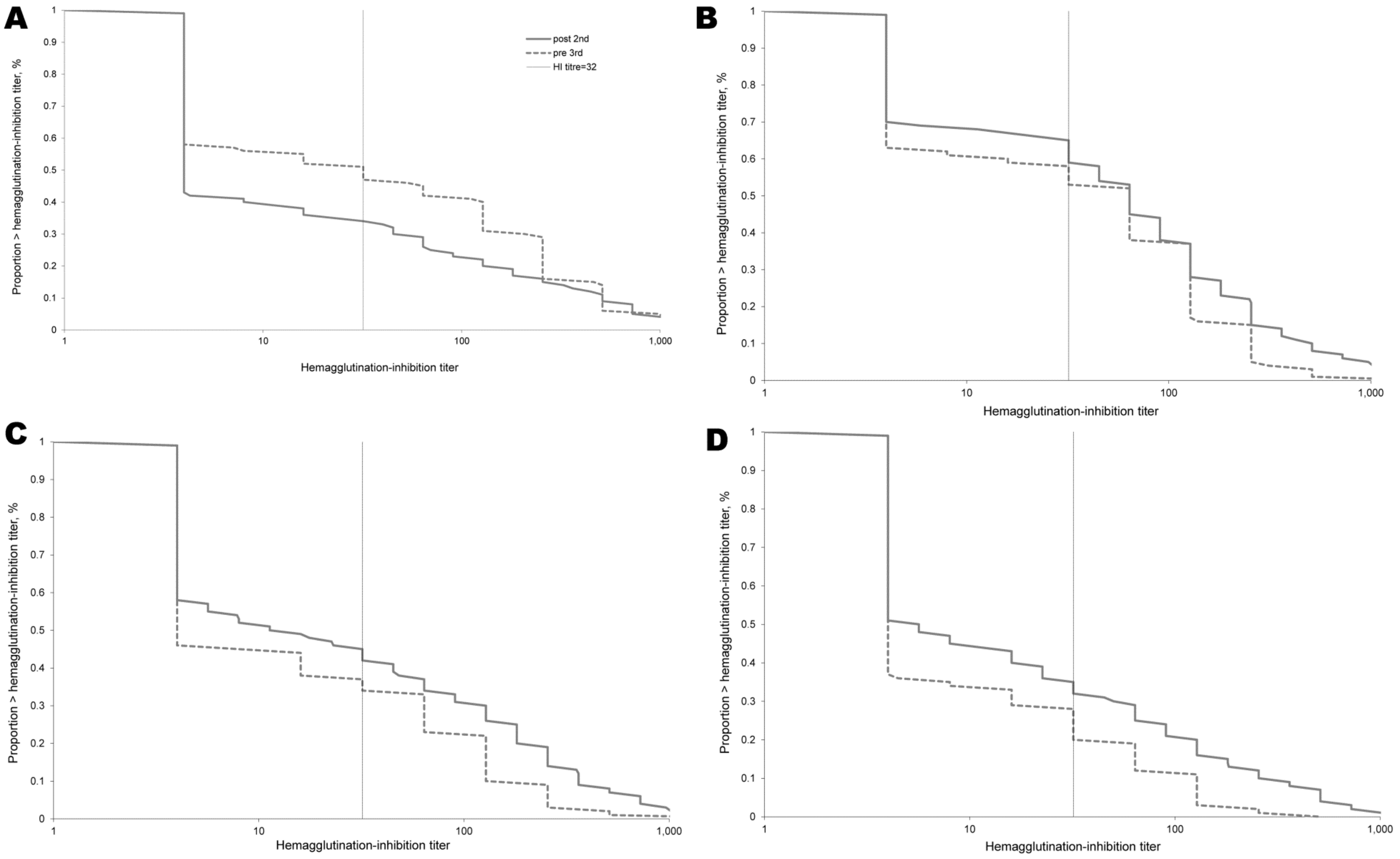
A
B
C
D
post 2nd
pre 3rd
HI titre=32
Proportion > hemagglutination-inhibition titer, %
Hemagglutination-inhibition titer
1
10
100
1,000
0
0.1
0.2
0.3
0.4
0.5
0.6
0.7
0.8
0.9
1

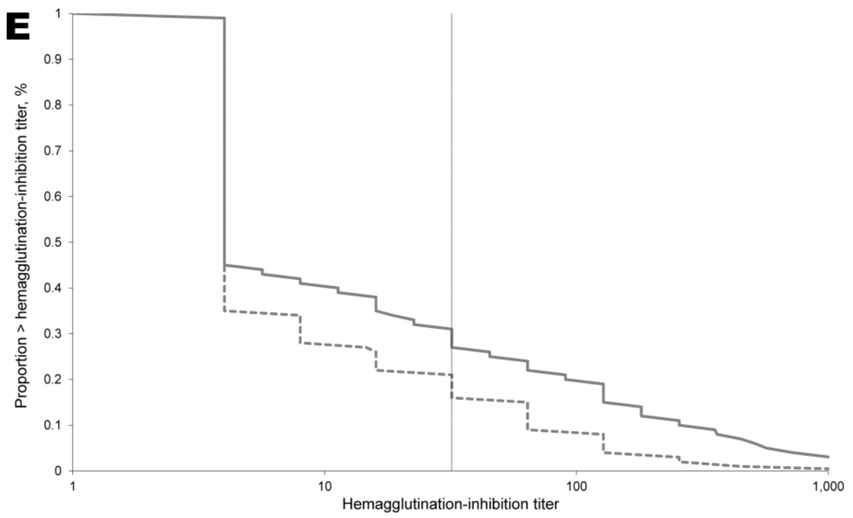
E
1
0.9
0.8
0.7
0.6
0.5
0.4
0.3
0.2
0.1
0
1
10
100
1,000
Proportion > hemagglutination-inhibition titer, %
Hemagglutination-inhibition titer

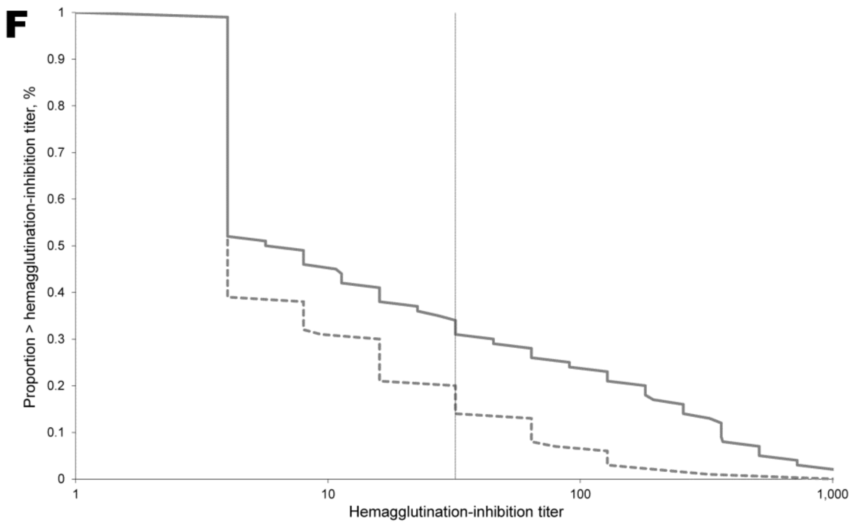
F
1
0.9
0.8
0.7
0.6
0.5
0.4
0.3
0.2
0.1
0
1
10
100
1,000
Proportion > hemagglutination-inhibition titer, %
Hemagglutination-inhibition titer

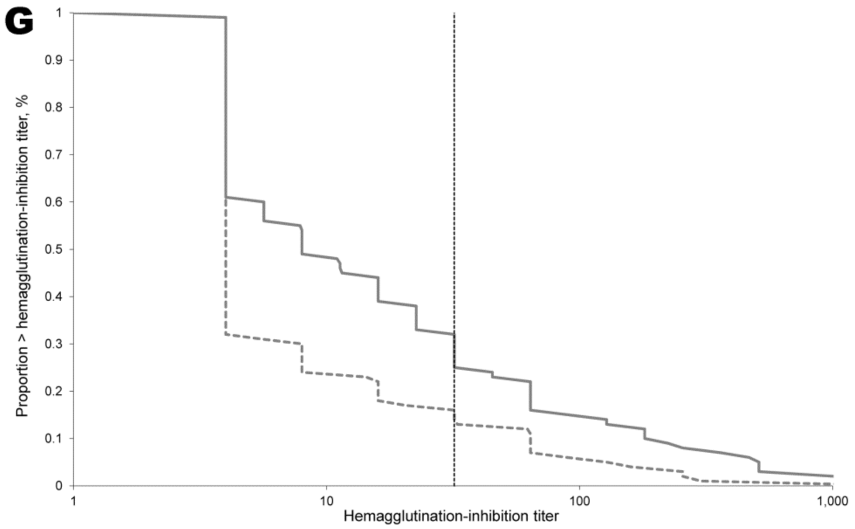
G
1
0.9
0.8
0.7
0.6
0.5
0.4
0.3
0.2
0.1
0
1
10
100
1,000
Proportion > hemagglutination-inhibition titer, %
Hemagglutination-inhibition titer
